# Inhibition Effect of Three-Dimensional (3D) Nanostructures on the Corrosion Resistance of 1-Dodecanethiol Self-Assembled Monolayer on Copper in NaCl Solution

**DOI:** 10.3390/ma11071225

**Published:** 2018-07-17

**Authors:** Shuai Hu, Zhenyu Chen, Xingpeng Guo

**Affiliations:** 1 Hubei Key Laboratory of Materials Chemistry and Service Failure, School of Chemistry and Chemical Engineering, Huazhong University of Science and Technology, Wuhan 430074, China; d201577113@hust.edu.cn; 2 School of Chemistry and Chemical Engineering, Guangzhou University, Guangzhou 510006, China

**Keywords:** copper, phosphoric acid, 3D nanostructures, 1-dodecanethiol SAM

## Abstract

A novel and simple method to improve the corrosion resistance of copper by constructing a three-dimensional (3D) 1-dodecanethiol self-assembled monolayer (SAM) in 3.5% NaCl solution is reported in this study. Several drops of 1% H_3_PO_4_ solution are thinly and uniformly distributed on copper surface to form a 3D nanostructure constituted by Cu_3_(PO_4_)_2_ nanoflowers. The anticorrosion properties of 1-dodecanethiol SAM on copper surface and on copper surface that is treated with H_3_PO_4_ solution were evaluated. Results demonstrated that 1-dodecanethiol SAM on bare copper surface exhibits good protection capacity, whereas a copper surface that is pretreated with H_3_PO_4_ solution can substantially enhance the corrosion resistance of 1-dodecanethiol SAM.

## 1. Introduction

Copper is widely used in microelectronic packaging due to its advantages, such as high electrical and thermal conductivities, low cost, and ease of manufacture [1]. Nevertheless, copper readily undergoes corrosion in practice.

In general, organic compounds containing nitrogen, oxygen, and sulfur, such as benzotriazole [2,3], 2-mercaptobenzothiazole [4], and 2-mercaptobenzimidazole [5], are frequently used as corrosion inhibitors for copper. In addition, self-assembled monolayers (SAMs) are widely used as barriers to protect copper against corrosion. These compact layers are constituted by highly ordered molecules and are formed spontaneously by chemisorption on a metal surface. Self-assembly has been recognized as a prospective technology for creating functional materials, owing to the extended and two-dimensional molecule layers that can provide excellent corrosion resistance and surface superhydrophobicity [6,7,8]. When compared with the traditional corrosion inhibition methods, SAMs exhibit the advantages of high coverage, few defects, and high inhibition efficiency [9,10]. Yamamoto et al. [11] reported that copper with alkanethiol self-assembled layers obtained excellent anticorrosion abilities. Alkanethiols were chemisorbed on the copper surface by covalent linking between Cu and S atoms, forming densely-packed, hydrophobic monolayers on the surface. Zhang et al. [12] studied the inhibition effect of Schiff base SAMs on copper. The maximum inhibition efficiency reached 93.9% for CO_2_-saturated simulative oilfield water after a 3 h self-assembly. Zhang et al. [13] revealed that ammonium pyrrolidine dithiocarbamate SAMs was a mixed-type inhibitor for copper in 3% NaCl solution, and sulfur atoms acted as the active adsorption sites during the self-assembly.

Reportedly, the protective properties of SAMs are closely related to chain length, packing density, number of defect, temperature, solvent, and the type of termination or anchoring groups [14,15,16,17]. Laibinis et al. [8] firstly reported that increasing the chain length of *n*-alkanethiolates retarded the oxidation of copper more obviously than that have shorter chain length, and an increase in the chain length of adsorbate by about 6 Å led to a corresponding decrease in the rate of oxidation by around 60%. Furthermore, Itoh et al. [18] demonstrated that further chemical modification of an 11-mercapto-1-undecanol SAM with alkyltrichlorosilanes improved the protective capability of monolayers against corrosion in aqueous and atmospheric environments. The anodic process of corrosion was inhibited by network structures, owing to two-dimensional polymerization with lateral siloxane linkage between molecules absorbed on copper.

In addition, electro-assisted methods, such as direct-current and alternation-current treatments, have been applied to further optimize self-assembled films [19,20,21]. Wang et al. [21] reported a novel method for fabricating an effective inhibition film on copper. Phenylthiourea was absorbed in a copper surface, 1-dodecanethiol was used for subsequent modification, and alternating-current voltage was applied on copper that was covered with the mixed film for further modification.

Structures and compositions of the metal surface markedly affect the protective property of SAMs. Inhomogeneity and thermodynamic instability of the metal substrate typically result in disordered adsorption layers and poor anticorrosion capability. Rohwerder et al. [22] reported that clean metal surface and metal surface that is covered with stable oxides were more beneficial for self-assembly than metal surface that is covered with unstable oxides. 

To date, few papers have reported on the effect of surface composition and structure on SAMs for the corrosion inhibition of copper. In this paper, the preliminary goal is the fabrication of stable nanostructures on the copper surface. According to previous research, several methods of constructing nanostructures on the copper surface were established. For example, Wang et al. [23] obtained desirable three-dimensional (3D) nanostructures on copper surface by immersing copper in H_3_PO_4_ solution. Structure and chemical composition were tunable by simply changing the concentration of H_3_PO_4_ solution and immersion time. He et al. [24] reported the Cu_3_(PO_4_)_2_ nanoflowers were formed through the interfacial reaction between copper foil and phosphate-buffered saline, and found that the formation of nanoflowers was related to the concentration of dissolved oxygen, chloride ions, and phosphate ions.

Herein, we reported a simple method for improving the corrosion resistance of SAM on copper via a pretreatment method using H_3_PO_4_ solution. 1-Dodecanethiol was selected for the formation of SAM. The copper surface was characterized using scanning electron microscopy (SEM), X-ray photoelectron spectroscopy (XPS), and X-ray diffraction (XRD). The corrosion resistance of 1-dodecanethiol SAM on H_3_PO_4_-treated copper surface was studied by using potentiodynamic polarization curves and electrochemical impedance spectroscopy (EIS).

## 2. Experimental

### 2.1. Materials and Solutions

Working electrodes were prepared from a copper sheet of purity 99.9%. For electrochemical studies, copper specimens were embedded in epoxy resin, with a surface area of 1 cm^2^ that was exposed to the electrolyte. The surface of the samples were initially ground with 400-grit emery paper and continued with 800- and 1200-grit emery papers successively. Then, the samples were washed with distilled water, degreased with ethanol and acetone, and finally dried with a flow of nitrogen gas.

1-Dodecanethiol (C_12_H_25_SH) from Aladdin with ≥98% purity was dissolved in absolute ethanol (AR grade) to a concentration of 80 g/L. An aqueous solution of 3.5% NaCl solution was prepared by dissolving NaCl (AR grade) in double-distilled water. H_3_PO_4_ (AR grade, ≥85%) concentrated solution was diluted to a concentration of 1%, and the pH value of the solution was adjusted to 2.5 by using NaOH solution.

### 2.2. Fabricating 3D Nanostructures on Copper Surface

50 µL of 1% H_3_PO_4_ solution was uniformly spread on the copper surface to form an ultrathin liquid membrane by rotating the electrode slightly. Then, the electrode was placed on a horizontal table and 15 µL of fresh 1% H_3_PO_4_ solution was added every 15 min to supplement the reactant and to maintain the volume of liquid membrane during the process. After 1 h, the sample was immersed in distilled water for 2 min to terminate the reaction. The temperature was controlled at 25 °C during preparation.

### 2.3. Formation of 1-Dodecanethiol SAM

The samples (copper and copper treated with H_3_PO_4_ solution) were dried in a vacuum dryer for 12 h to thoroughly remove water from the surface. The SAM was formed by immersing the samples in 1-dodecanethiol ethanol solution for 3 h. Then, the samples were washed with flowing distilled water and dried under nitrogen gas flow.

### 2.4. Characterization

Surface morphologies were obtained via scanning electron microscopy (SEM, Phillips Quanta 200 (FEI, Hillsboro, OR, USA) coupled with energy dispersive X-ray spectroscopy (EDS, FEI, Hillsborocity, OR, USA). XPS spectra were measured using a commercial VG Multilab 2000 system. An Al Kα radiation source (1486.6 eV) was equipped for the spectrum measurements and the electron energy resolution was 0.45 eV. Spectral decomposition was performed using background subtraction and a least-squares fitting program. XRD measurement was conducted on an Empyrean X-ray diffractometer (PANalytical B.V., Almelo, The Netherlands) from PANalytical (a Cu Kα irradiation source, *λ* = 1.5418 Å). The Attenuated Total Reflection Flourier transformed Infrared Spectroscopy (ATR-FTIR) was recorded using a Bruker VERTEX 70 Fourier transform infrared spectrophotometer (Bruker, Billerica, MA, USA) within a range of 4000 cm^−1^ to 400 cm^−1^. A narrow-band MCT detector (Bruker, Billerica, MA, USA), cooled with liquid nitrogen was used to detect the signal of bare copper with 1-dodecanethiol SAM.

Static contact angle measurements were carried out for the wettability evaluations of copper surface using the JC2000 contact-angle meter (Shanghai Zhongchen Digital Technic Apparatus Co., Ltd., Shanghai, China) at room temperature. Distilled water was used as solvent. A small water droplet was placed on the surface by using the microsyringe and the volume was controlled at 2 µL. The contact angle was determined by averaging the values that were obtained from three positions of each sample. The tilting angles were calculated using the Tangent method.

### 2.5. Electrochemical Measurements

Electrochemical measurements were conducted using an IM6e electrochemical workstation in a conventional three-electrode cell with 3.5% NaCl solution. Potentiodynamic polarization curves were obtained at a sweep rate of 0.5 mV/s in the potential range of −250 mV to +800 mV versus the open circuit potential. EIS measurements were conducted at the open circuit potential with a 10 mV amplitude perturbation at frequencies from 100 kHz to 10 mHz, with 10 points per decade. Impedance data were fitted to the appropriate equivalent circuits by using Zview software (3.0a Scribner Associates, Inc., Southern Pines, USA). All of the potential values in this paper refer to the saturated calomel electrode (SCE).

## 3. Results

### 3.1. Surface Characterization

#### 3.1.1. SEM Surface Morphologies

**Figure 1:**
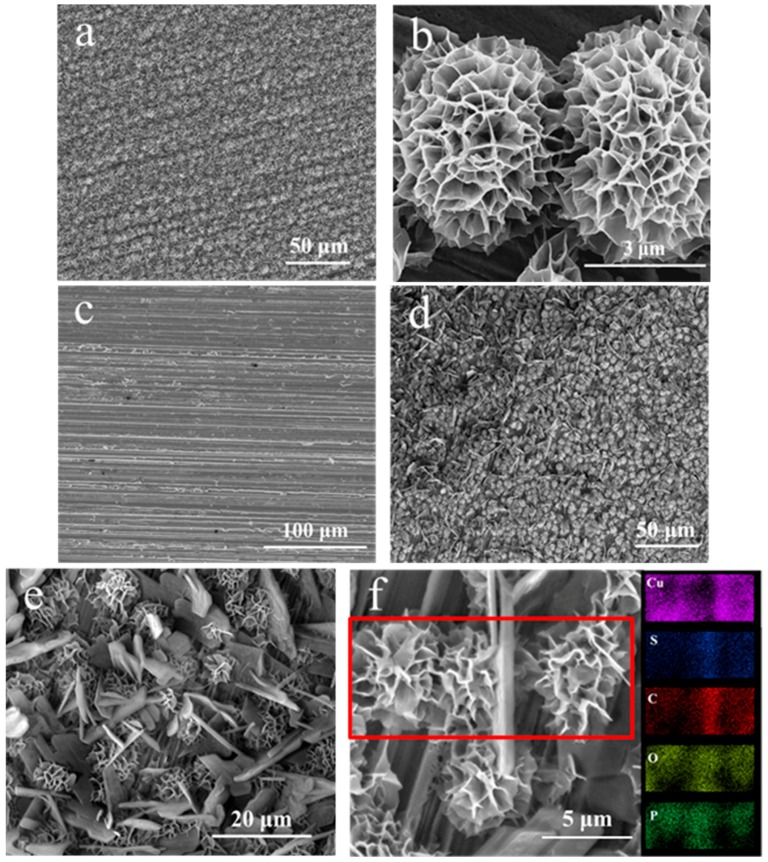

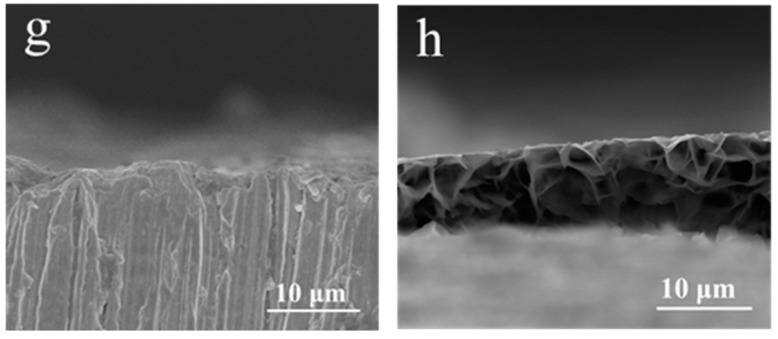
Scanning electron microscopy (SEM) images of (**a**,**b**) copper surface treated with H_3_PO_4_ solution; (**c**) copper surface with 1-dodecanethiol self-assembled monolayers (SAM); (**d**,**e**) copper surface-treated with H_3_PO_4_ solution and modified with 1-dodecanethiol SAM; (**f**) energy dispersive X-ray spectroscopy (EDS) mapping; (**g**) cross section of bare copper with 1-dodecanethiol SAM; and, (**h**) cross section of H_3_PO_4_ treated copper with 1-dodecanethiol SAM.

Figure 1a–f present the SEM pictures and EDS mapping for copper surface, Figure 1a,b treated with H_3_PO_4_ solution, Figure 1c with 1-dodecanethiol SAM, and Figure 1d–f with H_3_PO_4_ solution and modified with 1-dodecanethiol SAM. Figure 1g,h show the cross section of bare copper with 1-dodecanethiol SAM and H_3_PO_4_-treated copper with 1-dodecanethiol SAM. In Figure 1a, the copper surface is overspread with densely-packed flower-like nanostructures. In Figure 1b, the sheet structures of the free-standing Cu_3_(PO_4_)_2_ nanoflower can be observed clearly. Given that the surface was densely packed with these nanosheets, a 3D network full of channels and cavities was formed. Figure 1c shows the bare copper surface with 1-dodecanethiol SAM. In Figure 1d,e, the self-assembly of 1-dodecanethiol SAM on H_3_PO_4_-treated copper surface caused the formation of local multilayers of 1-dodecanethiol molecules that are distributed among the nanoflowers. According to the EDS mapping of the H_3_PO_4_-treated copper surface with 1-dodecanethiol SAM, the outline of the nanoflowers were observed clearly, and the chemical composition of the fragment is mainly C and S, which indicates that the fragments contain numerous 1-dodecanethiol molecules. In Figure 1g, the 1-dodecanethiol SAM on the surface is ultrathin; therefore, it is also invisible in the view of cross section. Figure 1h further illustrates the presence of numerous channels and cavities in the 3D network and it is beneficial for absorbing more 1-dodecanethiol molecules. The vague area in the picture is due the height difference between substrate and film.

#### 3.1.2. XPS Analysis

**Figure 2:**
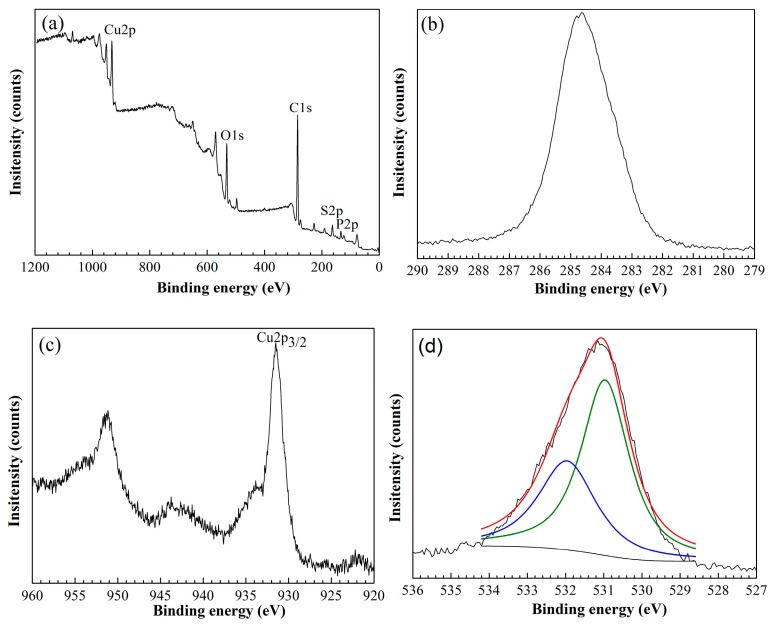

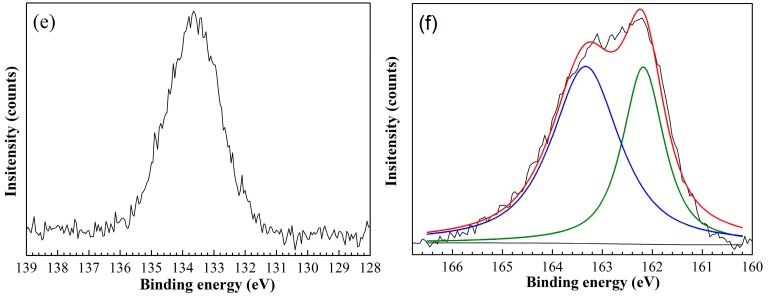
X-ray photoelectron spectroscopy (XPS) spectra of major elements on copper surface treated with H_3_PO_4_ solution and modified with 1-dodecanethiol SAM: (**a**) XPS; (**b**) C 1*s*; (**c**) Cu 2*p*; (**d**) O 1*s*; (**e**) P 2*p*; and (**f**) S 2*p*.

XPS analyses were carried out to determine the chemical composition of the copper surface. Figure 2a shows the XPS spectra of H_3_PO_4_-treated copper surface with 1-dodecanethiol SAM. The decomposition spectra for C, Cu, O, P, and S are shown in Figure 2b–f. The peak at 284.7 eV can be attributed to organic carbon and air contamination in the system [25]. A weak peak at 935.1 eV, which is characteristic of Cu_3_(PO_4_)_2_, was overlapped by the strong Cu 2*p*_3/2_ peak corresponding to Cu(I) or Cu(0) [26,27]. The XPS spectra of O 1*s* core level that decomposed into two components were located at 531.0 and 532.0 eV, which correspond to cuprous oxide and phosphate, respectively [28,29,30]. The peak at 133.6 eV in P 2*p* spectrum is attributed to P 2*p*_1/2_ of Cu_3_(PO_4_)_2_ [27]. The binding energy of S peak at 162.2 eV indicated that 1-dodecanethiol chemisorbed on the surface by thiolate formation, whereas the peak at 163.3 eV can be attributed to the presence of surface bound 1-dodecanethiol molecules [11,31,32].

Furthermore, surface analyses were implemented on the copper surface treated with H_3_PO_4_ solution and bare copper surface with 1-dodecanethiol SAM; the corresponding quantification (%) of each element is summarized in Table 1. From the Table, it is seen that the content of element S of bare copper surface with 1-dodecanethiol SAM is 2.03%, indicating the presence of 1-dodecanethiol molecules on the copper surface. Meanwhile, when compared with the chemical composition of bare copper surface with 1-dodecanethiol SAM, the contents of elements C and S of H_3_PO_4_-treated copper surface with 1-dodecanethiol SAM increased evidently. The results indicated that the 3D network of H_3_PO_4_-treated copper could accommodate more 1-dodecanethiol molecules by turning the two-dimensional (2D) SAM into 3D SAM.

#### 3.1.3. XRD Measurement

**Figure 3:**
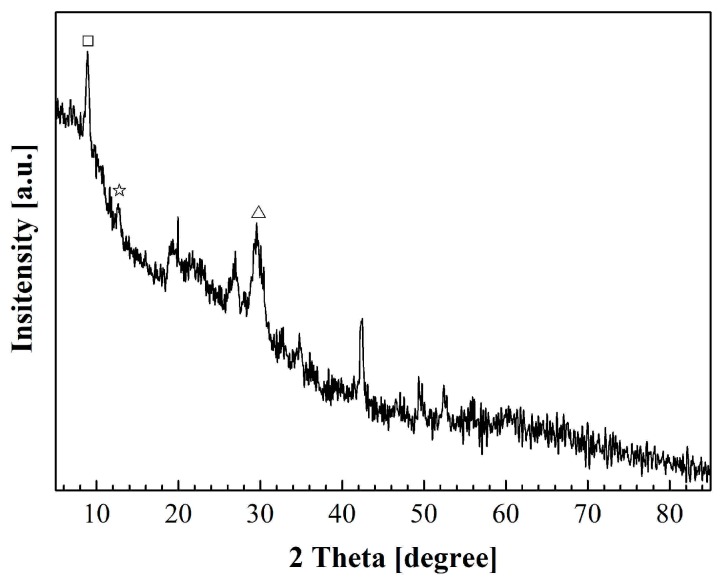
X-ray diffraction (XRD) pattern of Cu_3_(PO_4_)_2_ nanoflower.

The XRD pattern of the nanoflower is shown in Figure 3, and several peaks at 8.9°, 12.8°, and 29.5° match well with Cu_3_(PO_4_)_2_∙3H_2_O [JCPDS card 00-022-0548] [33]. These results revealed that the copper surface was covered with copper phosphate after H_3_PO_4_ solution treatment.

#### 3.1.4. FTIR Measurements

**Figure 4:**
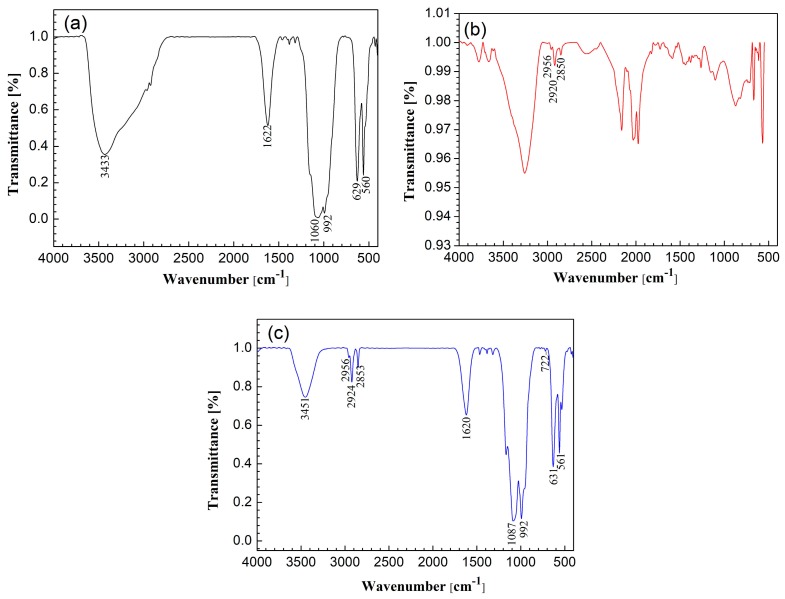
FTIR spectra of (**a**) copper surface treated with H_3_PO_4_ solution; (**b**) copper surface with 1-dodecanethiol SAM; and, (**c**) copper surface treated with H_3_PO_4_ solution and modified with 1-dodecanethiol SAM.

The FTIR spectra of copper surface treated with H_3_PO_4_ solution, pure 1-dodecanethiol, and copper surface treated with H_3_PO_4_ solution and modified with 1-dodecanethiol SAM are shown in Figure 4. The bands that appeared around 1625 cm^−1^ may be due to the absorbed water of Cu_3_(PO_4_)_2_ nanoflowers [24]. In Figure 4a, the bands at 1060 and 992 cm^−1^ correspond to the asymmetric and symmetric stretching vibrations of PO_4_^3−^ ions. The bands at 629 and 560 cm^−1^ are due to the out-of-plane bending vibrations of PO_4_^3−^ ions [24,34]. In Figure 4b, the band at 2956 cm^−1^ is assigned to the asymmetric stretching vibration of CH_3_. The bands at 2920 and 2850 cm^−1^ can be ascribed to the asymmetric and symmetric stretching vibrations of CH_2_, respectively [35]. The bands at 1464 cm^−1^ correspond to the bending vibration of S–CH_2_ [36]. Although slight shifts of these wave numbers were observed due to the disordered conformation of alkyl chains, the result was in good agreement with those of alkanethiol SAMs that are absorbed on gold [37,38,39]. Since the self-assembled monolayer on bare copper owned thickness of only a few nanometers and it was difficult to be detected in the FTIR measurement, the weak peaks in Figure 4b could be ascribed to the formation of 1-dodecanethiol multilayers [40]. The bands in Figure 4c match well with those in Figure 4a,b, indicating that 1-dodecanethiol molecules were strongly absorbed on H_3_PO_4_-treated copper surface.

#### 3.1.5. Contact Angle Measurements

**Figure 5:**
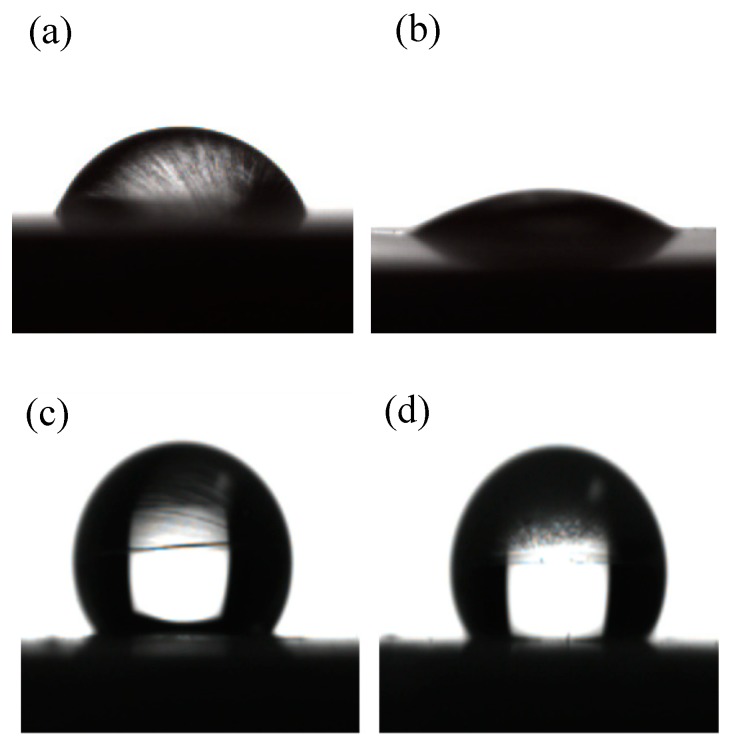
Contact angle of water droplets on (**a**) bare copper surface; (**b**) H_3_PO_4_-treated copper surface; (**c**) copper surface with 1-dodecanethiol SAM; and, (**d**) H_3_PO_4_-treated copper surface with 1-dodecanethiol SAM.

The wettability of 1-dodecanethiol SAM was tested by measuring the contact angle, which is closely linked to the adsorbed molecules, like odd-even chain length, terminal methyl group orientations, and order of SAM [35,41,42]. When the head of 1-dodecanethiol molecules were absorbed on the copper surface, densely-packed long alkyl chains were oriented towards the solution, resulting in a hydrophobic surface and increased contact angle. Figure 5 shows the water drop on the bare copper surface, H_3_PO_4_-treated copper surface, copper surface with 1-dodecanethiol SAM, and H_3_PO_4_-treated copper surface with 1-dodecanethiol SAM. The bare copper surface exhibited hydrophilicity with a water contact angle of approximately 71°, which was higher than expected, it could be the result of oxidation or contamination of the sample. Contact angles of bare copper surface treated with H_3_PO_4_, copper surface with 1-dodecanethiol SAM, and H_3_PO_4_-treated copper surface with 1-dodecanethiol SAM were 35°, 127°, and 125°, respectively. These results demonstrated that bare copper surface that is treated with H_3_PO_4_ became more hydrophilic, copper surface with 1-dodecanethiol SAM became hydrophobic, and the pre-treatment on copper surface with H_3_PO_4_ solution minimally affected the wettability of samples covered with 1-dodecanethiol SAM.

### 3.2. Corrosion Behaviors of Copper Treated with H_3_PO_4_ Solution

**Figure 6:**
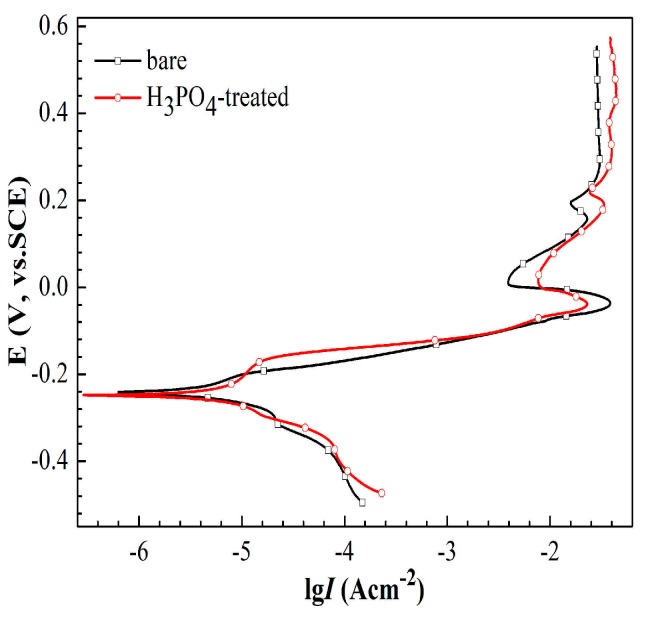
Potentiodynamic polarization curves of copper electrode with or without H_3_PO_4_ solution treatment.

Figure 6 shows the potentiodynamic polarization curves of copper electrode with or without H_3_PO_4_ solution treatment in 3.5% NaCl solution. The measurements were performed after the system was stabilized in NaCl solution for 30 min. The anodic reactions are generally considered to be reversible. The kinetics and mechanisms in the neutral NaCl solution are as follows [43]:

Cu + Cl^−^ ↔ CuCl + e^−^, (1)

CuCl + Cl^−^ ↔ CuCl_2_^−^, (2)

The typically anodic polarization curves for bare copper in neutral NaCl solution can be illuminated by dividing the curves into three parts according to the potential region. Section 1: From the Tafel region to the maximum current density. Section 2: a region of the current density decreased to the minimum. Section 3: a region of increasing in current density to the limited value. The increase in current density at Section 1 was due to the oxidation of copper to the cuprous ion. With the increase in potential, the decrease in current density to the minimum resulted from the formation of CuCl film. Then, CuCl was transformed to soluble CuCl_2_ at higher potentials, resulting in a limiting current density. Above the limiting-current region, the increase in current density might be caused by the formation of Cu(II) species. Moreover, the cathodic reaction can be related to the reduction of hydrogen ion or dissolved oxygen. Given that the equilibrium potential for hydrogen evolution in a solution at pH 7 is −662 mV versus SCE, the occurrence of hydrogen ion reduction is impossible. Correspondingly, the reduction potential of oxygen is 568 mV versus SCE, indicating that the reduction of oxygen is thermodynamically possible [44].

The corresponding potentiodynamic polarization parameters are listed in Table 2. The values of *i*_corr_ indicated that the bare electrode showed superior corrosion resistance than the H_3_PO_4_-treated electrode. This finding suggests that the 3D nanoflowers on the surface did not improve the corrosion resistance of copper without 1-dodecanethiol SAM modification.

**Figure 7:**
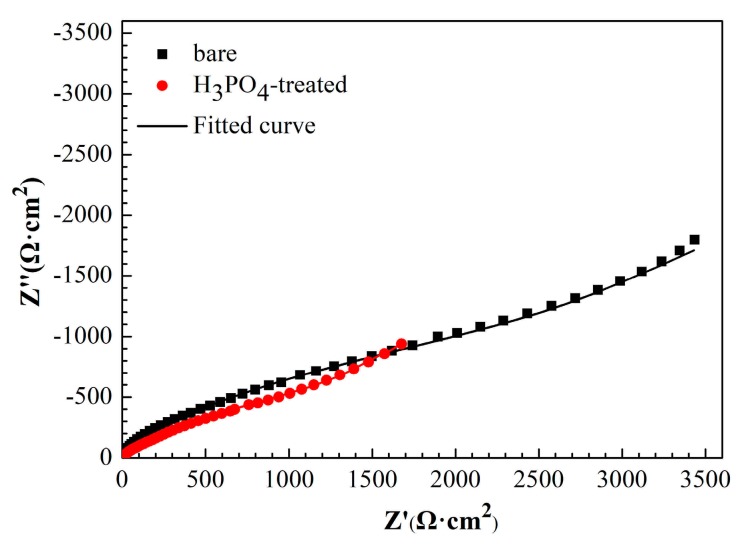
Nyquist plots of copper electrode with or without H_3_PO_4_ solution treatment.

**Figure 8:**
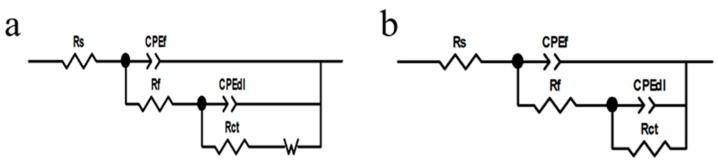
Equivalent circuits used for fitting impedance data: (**a**) bare copper electrode and copper electrode with 1-dodecanethiol SAM; (**b**) H_3_PO_4_-treated copper electrode with 1-dodecanethiol SAM.

Figure 7 shows the Nyquist plots of copper electrode with or without H_3_PO_4_ treatment in the 3.5% NaCl. EIS measurements were performed after the system was stabilized in NaCl solution for 30 min. Electrochemical impedance parameters were obtained using the equivalent circuit mode in Figure 8a and they are summarized in Table 3.

*R*_s_ is the solution resistance, *R*_f_ is the resistance of surface film, *R*_ct_ represents the charge-transfer resistance, W is the Warburg impedance, and CPE is the constant phase element. Given the lack of pure capacitance in the non-ideal electrochemical behavior of the surface, a CPE was used to substitute the pure capacitance. In general, a CPE is applicable to the case of surface inhomogeneity (e.g. electrode roughness and adsorbate) [45]. The impedance of CPE is defined, as follows:

*Z*_CPE_ = [*Y*(*jω*)*^n^*]^−^^1^, (3)

where *Y* is the magnitude of CPE, *ω* is the angular frequency, *j* is an imaginary number, and *n* is the exponential term represents the degree of the surface inhomogeneity.

Based on the parameters obtained from Table 3, H_3_PO_4_ treatment caused a negative influence on the corrosion resistance of copper, which is in accordance with the result of potentiodynamic polarization curves. According to the results of contact angle measurements, this could be attributed to the formation of more hydrophilic copper surface after the H_3_PO_4_ treatment.

### 3.3. Anticorrosion Investigations of 1-Dodecanethiol SAM

#### 3.3.1. Potentiodynamic Polarization Measurements

**Figure 9:**
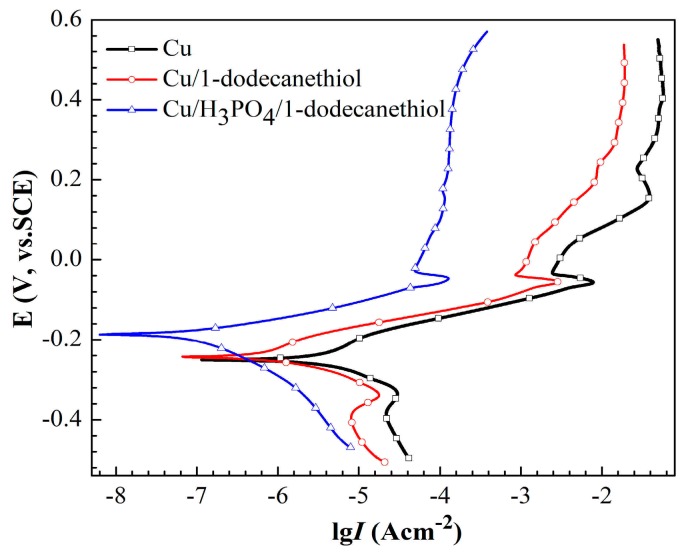
Potentiodynamic polarization curves obtained in 3.5% NaCl solution after 48 h for bare copper electrode, copper electrode with 1-dodecanethiol SAM, and copper electrode treated with H_3_PO_4_ solution and modified with 1-dodecanethiol SAM.

Figure 9 shows the potentiodynamic polarization curves that were obtained in 3.5% NaCl solution after 48 h immersion for bare copper electrode, copper electrode with 1-dodecanethiol SAM, and H_3_PO_4_-treated copper electrode with 1-dodecanethiol SAM. A small hump appeared on the cathodic curves for bare copper electrode and copper electrode with 1-dodecanethiol SAM at a potential around −300 mV versus SCE, which may be caused by the reduction of CuCl and/or Cu_2_O [30,46,47]. As seen from this image, the corrosion rate of bare copper electrode with 1-dodecanethiol SAM was effectively decreased. Meanwhile, the corrosion rate of H_3_PO_4_-treated electrode with 1-dodecanethiol SAM was decreased by one order of magnitude. In addition, the corrosion potential of the H_3_PO_4_-treated electrode with 1-dodecanethiol SAM notably shifted to a positive region.

The corresponding electrochemical results, such as corrosion potential (*E*_corr_), Tafel slope (*b*a, *b*c), and corrosion current density (*i*_corr_) are summarized in Table 4. Inhibition efficiency (IE %) were calculated using the following equation:

(4) $$\mathrm{IE}\;\%=\frac{i_{\mathrm{corr}}-i_{\mathrm{corr}}^{\prime }}{i_{\mathrm{corr}}}\times 100$$

where $i_{\mathrm{corr}}$ and $i_{\mathrm{corr}}^{\prime }$ are the corrosion current densities of bare copper electrode and electrodes with 1-dodecanethiol SAM in 3.5% NaCl solution, respectively. The inhibition efficiency increased from 73.1% to 97.2% when the electrode was processed with H_3_PO_4_ solution, which indicated that H_3_PO_4_ treatment of the copper surface prior to self-assembly is favorable for corrosion protection.

#### 3.3.2. EIS Measurements

**Figure 10:**
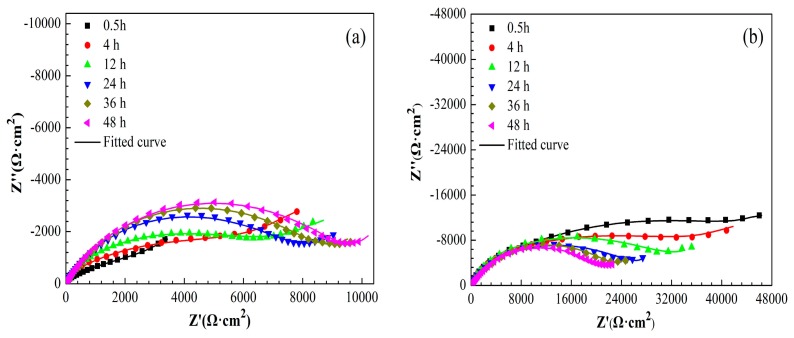

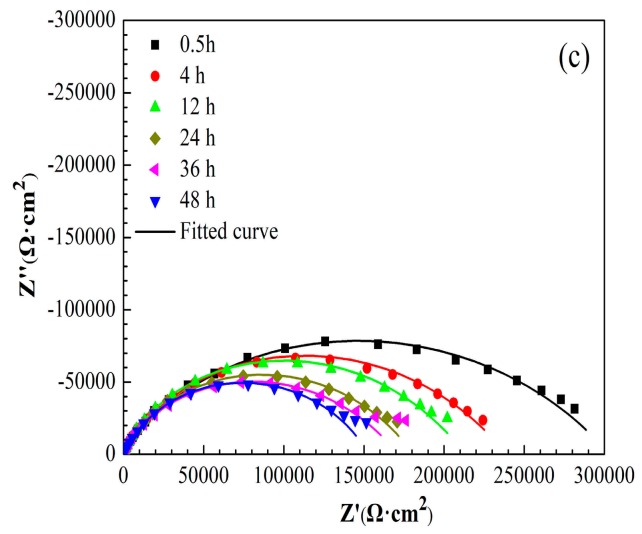
Nyquist plots of (**a**) bare copper electrode, (**b**) copper electrode with 1-dodecanethiol SAM, and (**c**) H_3_PO_4_-treated copper electrode with 1-dodecanethiol SAM in 3.5% NaCl solution at different times.

Figure 10 shows the Nyquist plots of bare copper electrode, copper electrode with 1-dodecanethiol SAM, and H_3_PO_4_-treated copper electrode with 1-dodecanethiol SAM in 3.5% NaCl solution at different times. The Nyquist plots of the bare copper electrode and copper electrode with 1-dodecanethiol SAM displayed a depressed semicircle at high frequency and a straight line in the low frequency region. The high-frequency semicircle could be attributed to the charge-transfer process, and the low-frequency linear portion namely, Warburge impedance was the result of the anodic diffusion of soluble CuCl_2_^−^ from the electrode/electrolyte interface to the bulk solution or the reduction of dissolved oxygen that is controlled by the oxygen diffusion [48,49]. The Warburg impedance in the Nyquist plot indicated that the corrosion of copper was influenced by the mass-transport process to a certain extent. Figure 10c shows that Warburg impedance disappears when the copper electrode was pre-treated using H_3_PO_4_ solution, indicating that corrosion was controlled by the charge-transfer process. The significant increase in the diameter of the arc could be ascribed to substantial increase of the charge transfer resistance that is caused by the formation of 1-dodecanethiol SAM on the H_3_PO_4_-treated copper surface, suggesting that the corrosion resistance was enhanced remarkably.

The electrochemical impedance parameters of the bare copper electrode and copper electrode with 1-dodecanethiol SAM was obtained while using the equivalent circuit mode in Figure 8a, whereas Figure 8b was used to fit those of the H_3_PO_4_-treated copper electrode with 1-dodecanethiol SAM. The corresponding values of these impedance parameters are listed in Table 5, Table 6 and Table 7.

As shown in Table 5, *R*_ct_ of bare copper increases with immersion time. This trend was relative to the formation of limited protective Cu_2_O and CuO films [44]. When compared with the bare copper electrode, the formed 1-dodecanethiol SAM on the bare copper surface considerably increased the *R*_ct_ values, indicating that 1-dodecanethiol SAM on the copper surface effectively inhibited corrosion. Furthermore, pretreatment of the copper electrode with H_3_PO_4_ solution can markedly enhance the corrosion resistance given that the *R*_ct_ value is one order of magnitude larger than that of the non-pretreated electrode. Corresponding to the increase of impedance values, the significant decrease in CPE_dl_ values for H_3_PO_4_-treated electrode can be ascribed to the replacement of water molecules by the adsorption of 1-dodecanethiol SAM on the electrode surface. The decrease in *R*_ct_ values with increasing immersion time was observed for both copper electrodes modified with 1-dodecanethiol SAM. This finding suggests that the 1-dodecanethiol SAM lose their protective property under continuous attack of dissolved oxygen and chloride ions.

## 4. Discussion

**Figure 11:**
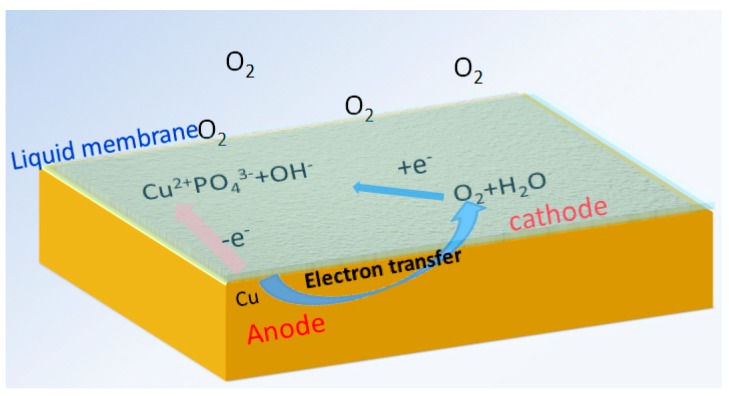
Schematic of the formation of Cu_3_(PO_4_)_2_ nanoflowers.

The pretreatment of copper surface with H_3_PO_4_ solution is necessary to obtain excellent protective properties. In our previous unsuccessful trials, the H_3_PO_4_ solution was placed on the copper surface without spreading, and the nanoflowers were found to grow on the margin of the liquid membrane on the copper surface, whereas nothing was found in the center, except for certain trails of corrosion. According to these results and those of previous reports [24,44], the formation of the Cu_3_(PO_4_)_2_ nanoflowers is closely linked to the concentration of dissolved oxygen and PO_4_^3−^ ions. The formation of Cu_3_(PO_4_)_2_ nanoflowers is illustrated in Figure 11, and the corresponding equations are as follows:

O_2_ + 2H_2_O + 4e^−^ → 4OH^−^ (cathodic reaction), (5)

Cu − 2e^−^ → Cu^2+^ (anodic reaction), (6)

Cu^2+^ + PO_4_^3−^ + 3H_2_O → Cu_3_(PO_4_)_2_∙3H_2_O, (7)

The released Cu^2+^ ions react with PO_4_^3−^ ions immediately, generating a solubilized layer of phosphate complex intermediate. The free phosphoric acid then corrodes the intermediate, resulting in selective crystallization into nanoparticles, which function as nuclei in the subsequent crystallization [23]. As the reactions progress, additional nanosheets are generated and aggregated into a flower-like sphere. Eventually, the surface is covered by the interconnected flower-like nanosheets. During the fabrication of nanoflowers, a paper-thin liquid membrane must be spread uniformly on the surface to ensure sufficient oxygen. Meanwhile, fresh H_3_PO_4_ solution is needed in order to maintain the liquid membrane and to supplement adequate PO_4_^3−^ ions.

**Figure 12:**
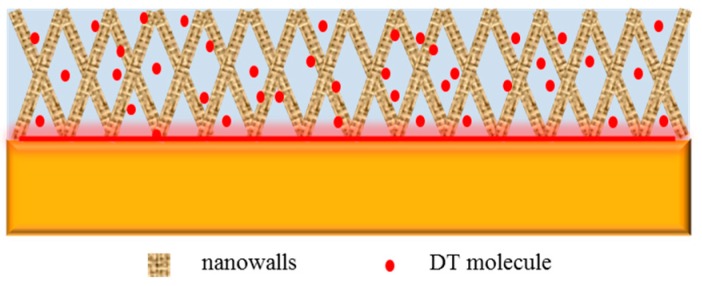
Schematic of networks on H_3_PO_4_-treated copper surface with 1-dodecanethiol SAM.

In the EIS measurements, it was found that 1-dodecanethiol SAM deteriorated with time. According to a previous report [50,51,52], 1-dodecanethiol SAM is sensitive to air exposure, which causes an increase in the density of defects and a decrease in the degree of order of the SAM. The mechanism for deterioration of 1-dodecanethiol SAM can be ascribed to the oxidation of thiolates to less-adherent sulfonates, leading to the roughening of the copper surface. The rougher surface may distort the structure of the hydrocarbon lattice and increase the permeability of the SAM [8,39]. When compared with the bare copper surface, copper surface treated with H_3_PO_4_ solution exhibited a large 3D network structure that adsorbed and accommodated additional 1-dodecanethiol molecules. Correspondingly, the 2D 1-dodecanethiol SAM on the bare copper was turned into 3D SAM. The interconnected nanosheets modified with 1-dodecanethiol monolayers constructed a 3D hydrophobic barrier to separate the substrate from the aggressive dissolved oxygen and chloride ions. Meanwhile, owing to the thick protective layer and the release of excess 1-dodecanethiol molecules in the cavities, the film exhibited increased durability in the NaCl solution. The schematic is shown in Figure 12.

## 5. Conclusions

A simple and novel method was performed to construct a three-dimensional 1-dodecanethiol SAM on copper surface. The corrosion resistance of 1-dodecanethiol SAM on bare copper surface and copper surface that is treated with H_3_PO_4_ solution was studied in 3.5% NaCl solution. Electrochemical measurements showed that the corrosion rate values of H_3_PO_4_-treated copper with 1-dodecanethiol SAM was one order of magnitude smaller than those of bare copper with 1-dodecanethiol SAM. Meanwhile, 1-dodecanethiol SAM deteriorated with the time under the attack of dissolved oxygen or chloride ions, which can be ascribed to an increase in the density of defects and a decrease in the degree of order of the SAM. From the results of SEM and FTIR, it was confirmed that a 3D network constituted of Cu_3_(PO_4_)_2_ nanoflowers was constructed on the copper surface and 1-dodecanethiol monolayer was self-assembled on the H_3_PO_4_-treated copper surface. The higher corrosion efficiency of H_3_PO_4_-treated copper with 1-dodecanethiol SAM can be attributed to the more absorption of 1-dodecanethiol molecules in the network and the formation of a three-dimensional barrier.

## Figures and Tables

**Table 1:** The atomic ratios (%) of elements on detected samples.

Sample	C	Cu	O	P	S
Cu/H_3_PO_4_	73.25	8.19	14.36	4.19	-
Cu/1-dodecanethiol	75.07	15.31	7.58	-	2.03
Cu/H_3_PO_4_/1-dodecanethiol	81.19	5.01	3.51	3.61	6.67

**Table 2:** Polarization parameters of copper electrode with and without H_3_PO_4_ solution treatment.

Sample	*E*_corr_ (mV)	*b*_a_ (mV/dec)	*b*_c_ (mV/dec)	*i*_corr_ (A/cm^2^)
Cu	−243	116	−60	6.60 × 10^−6^
Cu/H_3_PO_4_	−248	98	−66	7.81 × 10^−6^

**Table 3:** Electrochemical impedance parameters of copper electrode with and without H_3_PO_4_ solution treatment.

Sample	*R*_s_ (Ω·cm^2^)	CPE_f_ (S^n^·Ω^−1^·cm^−2^)	*n*	*R*_f_ (Ω·cm^2^)	CPE_dl_ (S^n^·Ω^−1^·cm^−2^)	*n*	*R*_ct_ (Ω·cm^2^)
Cu	3	1.96 × 10^−5^	0.96	81	2.94 × 10^−4^	0.47	3762
Cu/H_3_PO_4_	4	4.64 × 10^−5^	0.87	53	8.50 × 10^−4^	0.45	2649

**Table 4:** Polarization parameters of copper electrode, copper electrode with 1-dodecanethiol SAM and H_3_PO_4_-treated copper electrode with 1-dodecanethiol SAM in 3.5% NaCl solution for 48 h.

Sample	*E*_corr_ (mV)	*b*_a_ (mV/dec)	*b*_c_ (mV/dec)	*i*_corr_ (A/cm^2^)	IE%
Cu	−247	97	−66	4.75 × 10^−^^6^	-
Cu/1-dodecanethiol SAM	−235	199	−49	1.28 × 10^−^^6^	73.1
Cu/H_3_PO_4_/1-dodecanethiol SAM	−187	48	−205	1.32 × 10^−^^7^	97.2

**Table 5:** Electrochemical impedance parameters of bare copper electrode.

Sample	Time (h)	*R*_s_ (Ω·cm^2^)	CPE_f_ (S^n^·Ω^−1^·cm^−2^)	*n*	*R*_f_ (Ω·cm^2^)	CPE_dl_ (S^n^·Ω^−1^·cm^−2^)	*n*	*R*_ct_ (Ω·cm^2^)
Cu	0.5	3	1.96 × 10^−5^	0.96	81	2.92 × 10^−4^	0.47	3762
	4	4	2.62 × 10^−6^	0.98	293	8.18 × 10^−5^	0.46	7292
	12	4	4.23 × 10^−6^	0.98	109	5.98 × 10^−5^	0.51	7533
	24	4	4.18 × 10^−6^	0.98	137	4.20 × 10^−5^	0.68	7832
	36	4	4.04 × 10^−6^	0.95	155	4.09 × 10^−5^	0.71	8654
	48	4	3.73 × 10^−6^	0.92	185	3.51 × 10^−5^	0.69	9625

**Table 6:** Electrochemical impedance parameters of copper electrode with 1-dodecanethiol SAM.

Sample	Time (h)	*R*_s_ (Ω·cm^2^)	CPE_f_ (S^n^·Ω^−1^·cm^−2^)	*n*	*R*_f_ (Ω·cm^2^)	CPE_dl_ (S^n^·Ω^−1^·cm^−2^)	*n*	*R*_ct_ (Ω·cm^2^)
Cu/ 1-dodecanethiol SAM	0.5	6	1.69 × 10^−6^	0.88	6573	1.93 × 10^−5^	0.47	50,575
	4	5	2.00 × 10^−6^	0.88	6398	2.04 × 10^−5^	0.45	35,949
	12	7	2.46 × 10^−6^	0.98	4597	2.17 × 10^−5^	0.50	31,803
	24	6	3.08 × 10^−6^	0.94	3291	2.23 × 10^−5^	0.53	25,286
	36	4	3.96 × 10^−6^	0.89	1277	2.32 × 10^−5^	0.65	22,670
	48	4	5.04 × 10^−6^	0.92	931	2.52 × 10^−5^	0.64	21,026

**Table 7:** Electrochemical impedance parameters of H_3_PO_4_-treated copper electrode with 1-dodecanethiol SAM.

Sample	Time (h)	*R*_s_ (Ω·cm^2^)	CPE_f_ (S^n^·Ω^−^^1^·cm^−^^2^)	*n*	*R*_f_ (Ω·cm^2^)	CPE_dl_ (S^n^·Ω^−^^1^·cm^−^^2^)	*n*	*R*_ct_ (Ω·cm^2^)
Cu/H_3_PO_4_/ 1-dodecanethiol SAM	0.5	4	1.21 × 10^−6^	0.57	1086	1.04 × 10^−7^	0.88	302,811
	4	8	1.33 × 10^−6^	0.52	429	9.82 × 10^−7^	0.80	239,891
	12	5	1.41 × 10^−6^	0.52	347	1.21 × 10^−6^	0.84	214,319
	24	5	1.68 × 10^−6^	0.55	237	1.54 × 10^−6^	0.82	181,316
	36	5	1.86 × 10^−6^	0.50	202	1.79 × 10^−6^	0.81	172,380
	48	6	2.15 × 10^−6^	0.60	153	2.83 × 10^−6^	0.82	153,320
